# Omega-3 Supplementation in the Prevention of Contrast Induced Nephropathy in Patients Undergoing Elective Percutaneous Coronary Intervention: A Randomized Placebo-Controlled Trial

**DOI:** 10.15171/apb.2019.036

**Published:** 2019-06-01

**Authors:** Farzaneh Foroughinia, Mahtabalsadat Mirjalili, Ehsan Mirzaei, Alireza Oboodi

**Affiliations:** ^1^Clinical Neurology Research Center, Shiraz University of Medical Sciences, Shiraz, Iran.; ^2^Clinical Pharmacy Department, Shiraz University of Medical Sciences, Shiraz, Iran.; ^3^Student Research Committee, Shiraz University of Medical Sciences, Shiraz, Iran.

**Keywords:** Contrast Media, Creatinine, Cystatin C, Fatty acids, Omega-3, Percutaneous coronary Intervention

## Abstract

***Purpose:*** Contrast-induced nephropathy (CIN) is the third cause of hospital-acquired renal failure and is associated with significant morbidity and mortality. Several studies have revealed the protective role of omega-3 in prevention and treatment of some kidney injuries. This study was conducted to examine the effect of omega-3 supplementation on the markers of renal function and to evaluate its potential in the prevention of CIN in patients undergoing elective percutaneous coronary intervention (PCI).

***Methods:*** In this double-blind, randomized clinical trial, 85 eligible patients scheduled for PCI was randomly divided into omega-3 (a single 3750 mg dose of omega-3 as well as routine hydration therapy within 12 hours before PCI) or control (placebo plus routine hydration therapy) groups. Serum creatinine (SCr) and cystatin C levels were measured at baseline and 24 hours after PCI.

***Results:*** Our results indicated that post- PCI cystatin C levels were significantly decreased in the omega-3 group compared to the control group (*P* < 0.001). Although less upward manner was seen in the level of 24-hour creatinine in the omega-3 group, it did not reach the significance level (*P* = 0.008).

***Conclusion:*** The positive effect of omega-3 on cystatin C levels showed that it may have a protective role in the prevention of CIN in post-PCI patients with normal kidney function. However, to better assess this effect, it is highly recommended to design future studies with higher doses and longer duration of therapy with omega-3 plus long-term follow up.

## Introduction

Today, contrast agents have been increasingly administered for diagnostic and therapeutic purposes. However, they may result in clinical complications, such as contrast-induced nephropathy (CIN).^1^ CIN can lead to serious problems, including renal function impairment may be followed by a longer hospital stay, an increased rate of morbidity and mortality, and a higher financial cost.^2^ It accounts for about 11% of all hospital-acquired renal insufficiencies. Around 1%-2% of the general population and up to 50% of high-risk subgroups will experience this problem following coronary angiography (CA) or percutaneous coronary intervention (PCI).^3^ The renal insufficiency caused by receiving contrast agents during PCI may increase the risk of subsequent cardiac events and mortality in a dose-dependent manner during and after PCI even in patients with normal renal function.^4,5^ Nowadays, besides to serum creatinine (SCr), a new marker of renal function named cystatin C is used to assess renal status after PCI. It is a more sensitive and superior marker than SCr in the detection of acute kidney injury in the early stages of renal dysfunction.^6^

Although CIN can occur in any patient with exposure to contrast agents, some patients are more prone to encounter with this problem, such as patients with diabetes, hypertension, chronic heart failure, renal insufficiency, volume depletion, hemodynamic instability, older age, and those receiving hyperosmolal and higher doses of contrast media.^3,7,8^

Although different researches have been done on the prevention of CIN, no proven strategies exist till now. The official guideline published by the American College of Radiology suggests administration of prophylactic intravenous hydration in high-risk patients for CIN at least 6 hours before and after exposure to contrast media.^9^

Other pharmacologic precautions include minimization of the dose of contrast media and the use of iso-osmolar or low-osmolar contrast media. Furthermore, several studies have been conducted to assess the efficacy of different drugs, such as N-acetylcysteine (NAC), iloprost, alprostadil, prostaglandin E1, statins, bicarbonate sodium, ascorbic acid (vitamin C), vitamin E or its analogues (tocopherol), α-lipoic acid, atrial natriuretic peptide, B-type natriuretic peptide, and carperitide with/without hydration in the prevention of CIN.^10-14^

The mechanism of CIN is not fully understood. The suggestive mechanisms are direct toxicity of contrast media on the renal tubular epithelium, as well as inducing oxidative stress, inflammatory responses, ischemic injury, and renal tubular obstruction.^15^

Clinical studies have shown that omega-3 polyunsaturated fatty acids (PUFAs) can play an effective role in the reduction of inflammatory conditions in kidney diseases.^16^ It is reported that omega-3 have potential therapeutic effects for patients with renal disease and can improve kidney function.^17^ In a study, it was shown that omega-3 supplementation in diabetic patients with hypertriglyceridemia can decrease albuminuria and maintain renal function in a dose dependent manner.^18^

Although several studies reported the beneficial effects of omega-3 PUFAs in different kidney diseases,^18-20^ no randomized clinical trial have assessed its potential role in the prevention of CIN in the setting of coronary stenting, based on available databases. Therefore, we conducted this study to evaluate the efficacy of omega-3 supplementation in the prevention of CIN in patients undergoing elective PCI.

## Materials and Methods

A double-center, prospective, randomized double-blind clinical trial was performed in the cardiac catheterization laboratory of two tertiary care heart center affiliated to Shiraz University of Medical Sciences (SUMS) between May 2015 and June 2016. The trial was registered by the Iranian Registry of Clinical Trials ( identifier: IRCT2016041920441N4; https://www.irct.ir/). All participants signed an informed consent before being enrolled in the study.

### 

### Study population

Candidate patients for elective PCI with ages between 18 to 80 years old and an estimated glomeration filtration rate (eGFR) above 60 mL/min/1.73 m^2^ were eligible to be enrolled in this study. Patients were excluded if they had one of these excluding factors, including positive history of omega-3 supplementation on a regular basis in the last one month prior to admission; candidate for emergency coronary angioplasty; history of acute renal failure or end-stage renal failure requiring dialysis; history of heart bypass surgery in the last 3 months; history of allergy to aspirin, clopidogrel, and omega-3; report of unsuccessful PCI; positive signs of active bleeding; history of GI bleeding or peptic ulcer in the last one month; and history of NAC or vitamin C usage during last month.

eGFR was calculated using Cockcroft-Gault equation: GFR= (140-Age) x Body Weight (kg)/ 72 x Plasma Creatinine (mg/dL) (x 0.85 for female). Average of the pre-procedural creatinine during last 3 months before PCI, was applied for eGFR calculation.

### Study protocol

Based on institutional protocol, patients were admitted to the hospital at the day of PCI. A total of 88 patients were assigned to two groups by simple randomization. One and two patients in control and omega-3 groups, respectively, were excluded from the study as a result of withdraw of consent. Finally, 85 cases completed the trial (the power of study was estimated to be 99%). Patients received hydration therapy and either a single dose (3750 mg) of omega-3 in the omega-3 group or placebo (edible paraffin) in the control group within 12 hours before PCI. All participants underwent PCI by standard techniques and received single or multiple drug-eluting stent for various lesions. Nonionic agents, omnipaque and visipaque, were administered as contrast media.

The routine institutional treatment for the prevention of CIN consisted of hydration with normal intravenous (IV) 0.9% saline hydration alone (NaCl) in our center. Omega-3 pearls (SUPER NATURAL^®^; NUTRALAB, Canada) contained 1250 mg poly-unsaturated fatty acids, 600 mg eicosapentaenoic acid (EPA) and 300 mg docosahexaenoic acid (DHA), in each pearl. The administered omega-3 supplements lacked vitamin A. Placebo soft gels contained edible paraffine.

Patients’ demographic data, their relevant risk factors for CIN, type and volume of the contrast media, as well as medications and medical history of each patient were recorded. The Blood samples were collected from all patients at baseline and 24-hour after the intervention. In the omega-3 group, blood sampling was performed before receiving omega-3.

The SCr was measured using an automatic analyzer (Tajhizat Sanjesh Company, Iran) with reagents from Man Company (Cat. No. 101020) and the serum cystatin C concentration was determined by sandwich enzyme-linked immunosorbent assay (Human cystatin C quantikine ELISA kit, DSCTC0, R & D systems, USA). Laboratory data were obtained using the same laboratory and the same technique by staffs that were blinded to the study protocol.

### Study endpoints

The primary endpoint was the differences in post-PCI SCr and cystatin C between the study groups. Differences in pre/post-PCI creatinine and cystatin C among each group and the development of CIN were recorded as secondary endpoints. CIN was defined as a rise in the SCr concentration ≥0.5 mg/dL or 25% above baseline within 48-72-hour^3^ or a 10% or higher increase in cystatin C concentration within 24-h after exposure to contrast agent.^14^

### Statistical analysis

All data analysis was performed using the statistical package for social sciences version 21 (SPSS Inc, Chicago, USA). The Kolmogorov–Smirnov test was used to evaluate whether the variables were normally distributed which proved that the data distribution was non-normal. Categorical variables were presented as absolute and relative (percentage) frequencies. Continuous variables were expressed as mean ± standard deviation (SD). The independent samples *t* test was used to compare baseline and demographic parameters between study and control groups. The repeated measure ANOVA test was applied to compare the changes in the investigated markers from baseline to 24-hour follow-up between two groups. *P* values < 0.05 were considered significant.

## Results and Discussion

**Figure 1:**
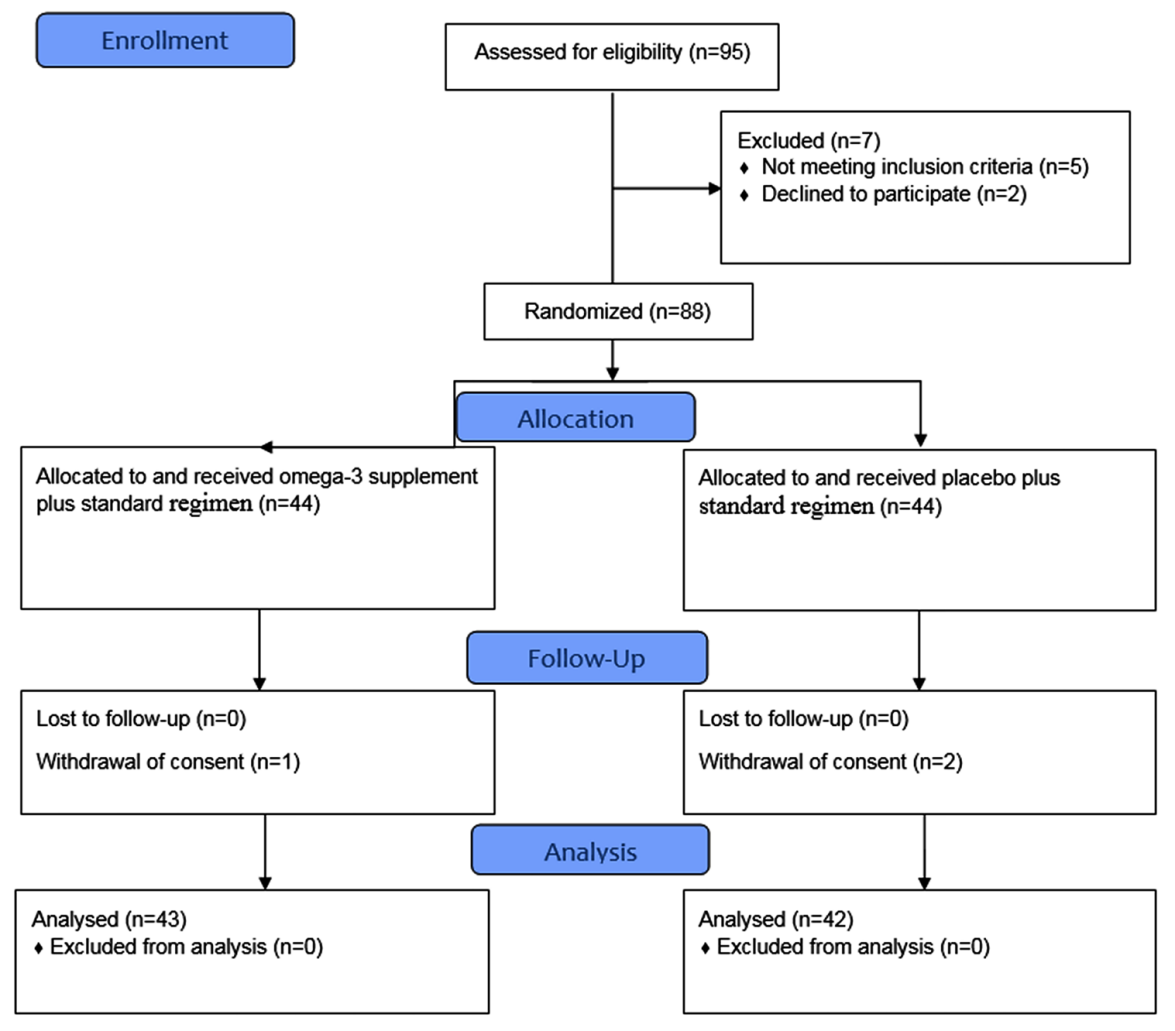
CONSORT trial flow diagram.
Legend: Randomized, double-blind, parallel-design, two-armed study (registered trial IRCT2016041920441N4). The 88 eligible subjects were randomly assigned to receive either omega-3 supplement plus routine hydration therapy (n = 44) or placebo plus routine hydration therapy (n = 44). The measurements and analysis were performed at 24 hours with 85 participants (n = 43 omega-3 group and n = 42 control group) while 3 subjects withdrew.

The CONSORT flow diagram of the clinical trial is shown in Figure 1. During the study period, a total number of 85 patients were recruited in the study, 43 and 42 cases in the omega-3 and control groups, respectively.

Demographic, clinical, and biochemical variables are reported in Table 1. The patients had the mean age of 56.7 ± 7.28 and 61.3±5.74 and gender distribution of 30 (71.4%) and 31 (72.1%) male in the control and omega-3 groups, respectively. There were no significant differences amongst groups except in age, history of hypertension, and beta-blocker consumption. No significant differences were observed between both groups in terms of the type and the volume of contrast agent (*P* = 0.847).

**Table 1:** Demographic data of the study and control groups

**Parameters**	**Omega-3 group (n = 43)**	**Control group (n = 42)**	***P*** **value**
Sex: Male, No. (%)	31 (72.1)	30 (71.4)	0.946
Age, year, mean ± SD	61.3 ± 5.74	56.7 ± 7.28	0.001
Weight, kg, mean ± SD	76.74 ± 12.93	78.66 ± 8.15	0.312
Diabetes mellitus, No. (%)	11 (25.6)	17 (40.5)	0.144
Smoker, No. (%)	7 (16.3)	8 (19)	0.738
Dyslipidemia, No. (%)	6 (14)	6 (14.3)	0.965
Hypertension, No. (%)	29 (67.4)	19 (45.2)	0.039
GFR	86.89 ± 10.02	94.71 ± 17.87	0.090
Used Medications‏
Statin, No. (%)	32 (74.4)	25 (59.5)	0.144
Beta Blockers, No. (%)	32 (74.4)	18 (42.9)	0.003
CCB, No (%)	11 (25.6)	12 (28.6)	0.756
ACEI and/or ARBs, No. (%)	28 (65.1)	25 (59.5)	0.595
Aspirin, No. (%)	24 (55.8)	31 (73.8)	0.083

SD, standard deviation; GFR, glomerular filtration rate; CCB, calcium channel blockers; ACEI, angiotensin converting enzyme inhibitors; ARBs, angiotensin receptor blockers.

**Figure 2:**
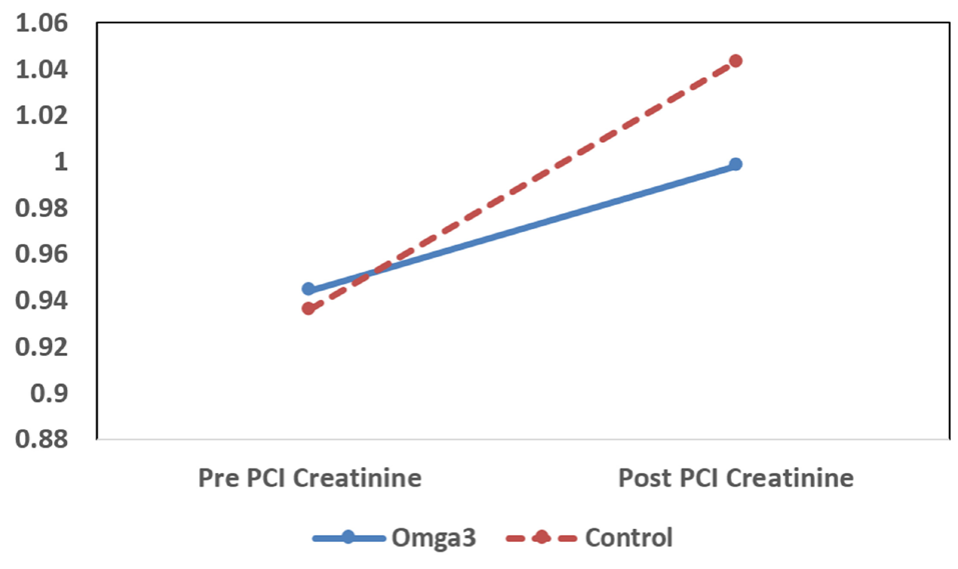
Comparison of mean serum creatinine concentrations in the two groups before and after percutaneous coronary intervention.

**Figure 3:**
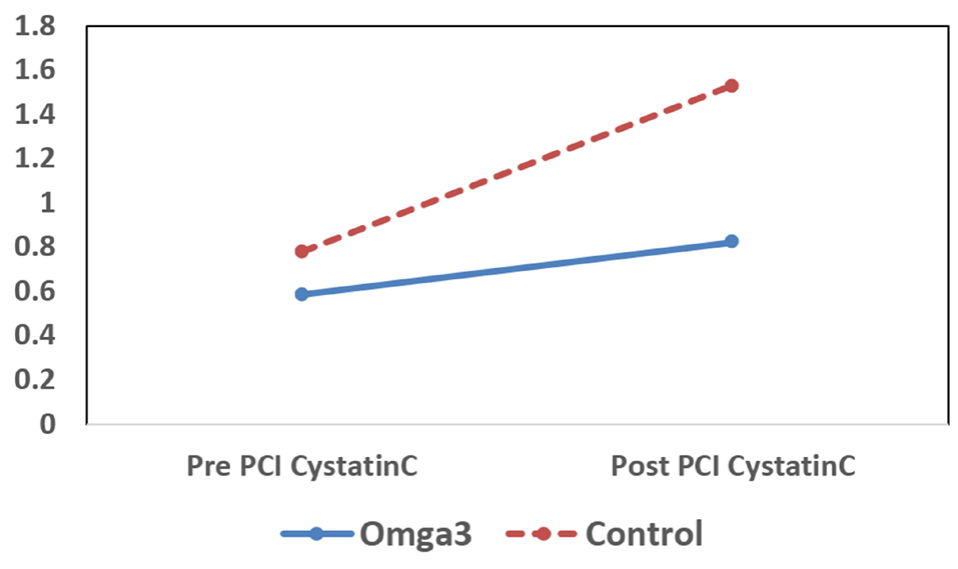
Comparison of mean cystatin C concentrations in the two groups before and after percutaneous coronary intervention.

The change in SCr and cystatin C level at baseline and 24 h after PCI for each study group is presented in Table 2. Average concentrations of SCr and cystatin C levels were significantly increased in both groups after PCI (*P *< 0.001) and this increase was more remarkable in the control group (Figures 2 and 3). However, on comparison between omega-3 and control groups, changes in SCr was not found to be statistically significant (*P *= 0.08). On the other hand, analysis of the data revealed that there was a significant difference between both groups in the changes of concentration of cystatin C level after PCI (*P *< 0.001). Therefore, treatment with omega-3 was more effective on the level of cystatin C than SCr.

**Table 2:** Mean baseline and follow up values for Serum Creatinine and Serum Cystatin C levels of test and control groups and results of Repeated measure ANOVA

**Variable**	**Group**	**Baseline**	**Follow up**	**Change**	***P*** **value**
		**Mean ± SD**	**Mean ± SD**		
Serum creatinine	Omega-3 group	0.944 ± 0.085	0.998 ± 0.128	0.054	0.08
	Control	0.936 ± 0.132	1.043 ± 0.159	0.107	
	Total	0.940 ± 0.1104	1.020 ± 0.145	0.080	
Serum cystatin C	Omega-3 group	0.588 ± 0.353	0.824 ± 0.593	0.136	<0.001
	Control	0.781 ± 0.388	1.535 ± 0.612	0.754	
	Total	0.683 ± 0.381	1.175 ± 0.697	0.492	

Evaluation of patients’ cystatin C markers did not show any CIN occurrences in the two groups. On the other hand, evaluation of creatinine showed one case of CIN occurrence in the omega-3 group (1.1%). Data analysis via the chi-square test revealed that no significant difference was observed between the two groups with regards to the occurrence of CIN (*P* = 0.506). Meanwhile, no significant difference was observed between the mean of age, weight and GFR between this patient and the rest of patients who were not afflicted by CIN.

According to the available literatures, this trial is the first randomized study evaluating the potential role of omega-3 supplement, as an antioxidant, in the prevention of CIN in patients treated with PCI.

CIN is an important medical issue, since it may lead to medical problems, such as acute renal failure, prolonged hospital stays, consequent complications, increased mortality rate, as well as higher medical cost. It has reported that even small increases in SCr and cystatin C levels caused by intravascular radiocontrast administration after CA are associated with adverse outcomes.^21,22^

Up to now, numerous agents have been studied for the prevention of CIN. Despite strong logic behind the implementation of these substances, most of them were not that efficient. Hopefully, reported clinical trials provided relatively acceptable results in the use of antioxidants, such as N-acetylcysteine and ascorbic acid, as well as intravenous fluids including sodium bicarbonate in this setting.^10-14^

Several studies have shown that supplementation with EPA and DHA, essential fatty acids of omega-3, can attenuate inflammatory diseases, including myocardial infarction.^23-28^ There are evidences that omega-3 fatty acids are capable of being used as adjunctive therapies in specific kidney diseases, such as Immunoglobulin A (IgA) nephropathy, chronic renal diseases, dialysis and renal cancers.^29^ Possible mechanisms suggested for the positive effects of omega-3 in the prevention of chronic kidney diseases are increasing the amount of eicosanoids and the endothelium derived relaxing factor in the blood, as well as decreasing the amount of inflammatory cytokines, such as IL-6, IL-1ra, TNF-alpha, sIL-6r and TGF-beta, blood pressure, serum triglycerides, and platelet aggregation.^30-34^ As a result, in this study, omega-3 was selected as the targeted drug due to its reported positive effects on renal and cardiovascular complications.

In an animal study, it was concluded that preventive administration of omega-3 in renal reperfusion injuries in rats could lead to a decrease in renal dysfunction, oxidative stress and histological damages. These positive outcomes may be resulted from the effects of omega-3 in augmenting the level of anti-inflammatory markers and protective factors in the kidney, reducing pre-inflammatory factors, and limiting the access to arachidonic acid; therefore, preventing the consequential inflammatory process.^35^ Previous studies have stated that long-term use of unsaturated omega-3 fatty acids could improve renal function and reduce the risk of developing advanced stages of kidney disease. For instance, EPA is capable of producing positive effects in patients with lupus nephritis by reducing the oxidative stress.^36^ One study mentioned that daily consumption of 1.86 g EPA and 1.5 g DHA for one year could suppress progression of albuminuria in subjects with type 2 diabetes mellitus and coronary artery disease.^37^ Another study revealed that using omega-3 in patients with diabetes and hypertriglyceridemia could prevent the progression of diabetic nephropathy and preserved GFR in a dose dependent manner.^18^ Omega-3 can be effective in renal diseases, even in low doses; as it was shown in a clinical trial in which administration of 0.85 g EPA and 0.57 g DHA slowed progression of kidney dysfunction in high-risk patients with IgA nephropathy and particularly those with advanced renal disease.^38^

In our study, less upward manner in the levels of both SCr and cystatin C were seen in the omega-3-treated patients, therefore; the addition of omega-3 to routine hydration therapy may be effective in the prevention of CIN in patients undergoing PCI. In contrast to creatinine, statistically significant difference was noted between the omega-3 and control groups in the terms of serum cystatin C. Although the benefit of treatment with omega-3 in reducing cystatin C did not translate to clinical outcome including significant decrease in CIN occurrence, still it may be of value in the prevention of renal dysfunction in the late phase after PCI similar to the effect of drugs, such as angiotensin-converting enzyme inhibitors.^39^

Despite randomization, factors such as age and history of hypertension were statistically different between two groups; means patients in the omega-3 arm were older and had more history of hypertension than control group. Thus, to remove the cofounding effect of these variables, the repeated measure ANOVA test was used. Our analysis reported that the difference in age between study groups did not have any significant effect on kidney markers studied in this trial (*P* = 0.222 for creatinine, and *P* = 0.133 for cystatin C). On the other hand, our results showed that in normotensive and hypertensive patients, the rise of cystatin C level after PCI was significantly less in omega-3 group in comparison to control group (*P* < 0.001 in both hypertensive and normotensive patients) (Table 3). In other words, treatment with omega-3 was significantly effective on cystatin C levels regardless of hypertension. But considering SCr level, it was observed that our intervention was significantly more effective in patients without hypertension (*P *= 0.029), in compare to patients with the history of hypertension (*P *= 0.356) (Table 3). This result was not out of expectation since hypertension is one of the underlying risk factors affecting renal function following cardiac procedures and some studies showed that prior history of hypertension could be a good predictor of CIN.^40^

**Table 3:** Comparison of Mean baseline and follow up values for Serum Creatinine and Serum Cystatin C levels of test and control groups and results of Repeated measure ANOVA in patients with and without HTN

	**Variable**	**Group**	**Baseline**	**Follow up**	**Change**	***P*** **value**
			**Mean ± SD**	**Mean ± SD**		
Without HTN	Serum creatinine	Omega-3 group	0.959 ± 0.650	0.956 ± 0.085	-0.003	0.029
		Control	0.926 ± 0.138	1.026 ± 0.165	0.1	
		Total	0.935 ± 0.115	1.003 ± 0.142	0.068	
	Serum cystatin C	Omega-3 group	0.634 ± 0.397	0.761 ± 0.655	0.127	<0.001
		Control	0.794 ± 0.304	1.569 ± 0.562	0.775	
		Total	0.733 ± 0.346	1.264 ± 0.711	0.531	
With HTN	Serum creatinine	Omega-3 group	0.941 ± 0.945	0.998 ± 0.128	0.057	0.356
		Control	0.947 ± 0.126	1.013 ± 0.143	0.066	
		Total	0.943 ± 0.107	1.033 ± 0.147	0.09	
	Serum cystatin C	Omega-3 group	0.565 ± 0.335	0.854 ± 0.571	0.289	0.004
		Control	0.765 ± 0.479	1.493 ± 0.670	0.728	
		Total	0.644 ± 0.405	1.107 ± 0.686	0.463	

HTN, Hypertension; SD, Standard deviation.

This study had several limitations. The major limitation was that relatively few patients were evaluated. Second, patients were treated with a single dose of omega-3, while treating for longer period of time may reveal the positive effect of omega-3 much better. Third, the patient follow-up period was just 24 hours after PCI, while 72-hour SCr is more accurate than 24-h sample in the assessment of CIN. Since patients were discharged 24 hours after PCI according to institutional program and most of them were from other cities, it was not possible to have their 72-hour blood sample for measuring the investigated markers.

## Conclusion

The positive effect of omega-3 on cystatin C levels showed that it may have a protective role in the prevention of CIN in post-PCI patients with normal kidney function. However, to better assess this effect, it is highly recommended to design future studies with higher doses and longer duration of therapy with omega-3 plus long-term follow up.

## Ethical Issues

Ethical approval was obtained from the ethics committee of the Shiraz University of Medical Sciences (ethics code: IR.SUMS.REC.1394.208).

## Conflict of Interest

The Authors declare that there is no conflict of interest.

## Acknowledgments

This research, extracted from a thesis written by Alireza Oboodi, was financially supported by Pharmaceutical Sciences Research Center, Shiraz University of Medical Sciences (Grant number: 8323).

The authors would like to express their gratitude to Center for Development of Clinical Research of Nemazee Hospital and Dr. S. T. Heydari for statistical assistance and also to managers and staffs of Kowsar and Alzahra hospitals for their support.
